# The Pharmacological Properties and Therapeutic Use of Apomorphine

**DOI:** 10.3390/molecules17055289

**Published:** 2012-05-07

**Authors:** Samo Ribarič

**Affiliations:** 1 Institute of Pathophysiology, Medical Faculty, University of Ljubljana, Zaloška 4, SI-1000 Ljubljana, Slovenia; Email: samo.ribaric@mf.uni-lj.si; Tel.: +386-1-543-70-20; Fax: +386-1-543-70-21; 2 Laboratory for Movement Disorders, Department of Neurological Disorders, University Clinical Centre Ljubljana, Zaloška 2, SI-1000 Ljubljana, Slovenia

**Keywords:** apomorphine, Alzheimer’s disease, dopamine agonist, erectile dysfunction, Parkinson’s disease

## Abstract

Apomorphine **(**APO) is an aporphine derivative used in human and veterinary medicine. APO activates D_1_, D_2S_, D_2L_, D_3_, D_4_, and D_5_ receptors (and is thus classified as a non-selective dopamine agonist), serotonin receptors (5HT_1A_, 5HT_2A_, 5HT_2B_, and 5HT_2C_), and α-adrenergic receptors (α_1B_, α_1D_, α_2A_, α_2B_, and α_2C_). In veterinary medicine, APO is used to induce vomiting in dogs, an important early treatment for some common orally ingested poisons (e.g., anti-freeze or insecticides). In human medicine, it has been used in a variety of treatments ranging from the treatment of addiction (*i.e.*, to heroin, alcohol or cigarettes), for treatment of erectile dysfunction in males and hypoactive sexual desire disorder in females to the treatment of patients with Parkinson's disease (PD). Currently, APO is used in patients with advanced PD, for the treatment of persistent and disabling motor fluctuations which do not respond to levodopa or other dopamine agonists, either on its own or in combination with deep brain stimulation. Recently, a new and potentially important therapeutic role for APO in the treatment of Alzheimer’s disease has been suggested; APO seems to stimulate Aβ catabolism in an animal model and cell culture, thus reducing the rate of Aβ oligomerisation and consequent neural cell death.

## 1. Introduction

Apomorphine (APO), an aporphine derivative of the dibenzoquinoline class, is used in human and veterinary medicine. It can be produced by heating morphine in an acid solution (e.g*.*, with hydrochloric acid) or alternatively synthesized from other starting materials [1]. APO is very lipophilic and moderately soluble in water and normal saline. Oxidation upon exposure to air and light is rapid, but can be prevented by the use of strong acids (e.g*.*, hydrochloric acid or zinc chloride) and antioxidants [2].

APO activates D_1_-like (D_1_, D_5_) and D_2_-like (D_2_, D_3_, D_4_) receptors (and is thus classified as a non-selective dopamine agonist), serotonin receptors (5HT_1A_, 5HT_2A_, 5HT_2B_, and 5HT_2C_), and α-adrenergic receptors (α_1B_, α_1D_, α_2A_, α_2B_, and α_2C_) [1,3]. APO’s dopaminergic activity is due to its polycyclic and tertiary amine structures. A relatively weak, inhibitory action of APO on presynaptic dopamine receptors has been suggested as an explanation of why low or declining APO plasma levels sometimes lead to a clinical worsening, either before the onset of clinical effects or at the end of an improved motor response in Parkinson’s disease (PD) [4].

In addition to its receptor mediated actions, APO is a highly potent antioxidant and free radical scavenger with a neuroprotective effect that has been demonstrated *in vitro* and *in vivo* [5,6]. Recent studies suggest a possible, non-receptor mediated neuroprotective effect in an animal model of Alzheimer’s disease (AD, see Section 3.3). However, some neuroprotective effects are also receptor-mediated. For example, APO treatment of astrocyte cultures stimulated biosynthesis and extracellular release of basic fibroblast growth factor 2. The astrocyte-derived conditioning medium improved, in a dose-dependent manner, the survival rate of the recipient tyrosine hydroxylase-positive neurons [7]. To date, there is no evidence that APO is neuroprotective in humans with PD.

APO has a very rapid onset of action combined with a brief duration of effect [8]. For example, patients with PD have reported an improvement of motor function three minutes after intravenous (*i.v*.) administration of a single dose, or five minutes after subcutaneous (*s.c.*) bolus administration. The duration of APO action, after a single administration, is dose and mode of administration dependent, with an elimination half-life ranging from 30 to 90 min [8]. The drug absorption, volume of distribution, plasma clearance, and half-lives are similar for *s.c.* injection, *s.c* infusion, and *i.v*. infusion, although it should be noted that the variation in absorption is high between subjects but low within individual subjects [8]. Many routes of APO administration have been used in clinical practice, but *s.c.*, sublingual (*s.l.*), nasal and rectal administration are preferred and *i.v*. administration should be avoided since it can cause crystallization of the drug, leading to thrombus formation and pulmonary embolism [9]. Oral administration is not recommended because of APO’s significant first-pass hepatic metabolism and poor bioavailability. For example, early clinical use of orally administered APO in patients with PD was abandoned, in favor of levodopa with a higher efficacy/safety ratio, because the very large APO doses often precipitated adverse effects like nausea, postural hypotension and prerenal azotemia due to the accumulation of a hepatically produced nephrotoxic metabolite and because of the drug’s short duration of action [10]. APO is very lipophilic and equilibrates rapidly between blood and tissue compartments. High lipid solubility leads to transient APO brain concentrations which can be up to eight-times higher than those in plasma [8]. However, clinical studies suggest that the pharmacodynamic effects of APO last up to 30 min after the plasma concentration of APO falls below the peak ineffective threshold [11]. The elimination of APO from plasma, reviewed by Gancher *et al.* 1989 [8], can be described either by a one-compartment or by a two-compartment model. APO is a high clearance drug (3 to 5 L/kg/h) that is mainly excreted and metabolized by the liver. Glucuronidation and sulfation are both responsible for about 10% of APO’s metabolic transformation. Because of significant systemic oxidation, less than 5% of APO is excreted unchanged in the urine [1,12].

The sedative-hypnotic properties of APO have been known since the end of the 19th century and the drug was used to treat insomnia, depression or schizophrenia. In the 20th century, APO was used in a variety of treatments, from the treatment of addiction (*i.e.*, to heroin, alcohol or cigarettes) to the treatment of Parkinson’s disease. APO was the first dopamine receptor agonist used to treat PD patients, with the first documented clinical report being published in 1951. However, as mentioned above the disadvantages of oral APO administration (peripheral dopaminergic adverse effects and short duration of action) led to its replacement with levodopa. In the mid 1980s, the introduction of alternative modes of APO administration that bypass first-pass liver metabolism (e.g*.*, subcutaneous or sublingual) renewed interest in the drug. The use of APO for treatment of erectile dysfunction in males was systematically evaluated in the first decade of the 21st century [10,13]. 

Currently, APO is mainly used in patients with advanced PD, for the treatment of persistent and disabling motor fluctuations which do not respond to levodopa or other dopamine agonists, either on its own or in combination with deep brain stimulation [14]. Recently, a new and potentially important therapeutic role for APO in the treatment of Alzheimer’s disease (AD) has been suggested; APO seems to stimulate Aβ catabolism, thus reducing the rate of Aβ oligomerisation and consequent neural cell death. In veterinary medicine, APO is also used to induce vomiting in dogs [15], an important early treatment of poisoning, after ingestion of a toxic compound (e.g., anti-freeze or insecticide).

## 2. Apomorphine Treatment in Parkinson’s Disease

Parkinson’s disease is a common neurodegenerative disease (second only to Alzheimer’s disease) affecting 1 in 500–700 people. The cause of PD disease is known in only a small number of patients suffering the hereditary form. Therefore, the majority of patients have the idiopathic form of PD. Whatever the cause, the clinical signs of PD, *i.e.*, parkinsonism, are a reduction in the frequency and speed of movement (hypokinesia), tremor and muscle rigidity with difficulty of walking and gait. Autonomic dysfunction, cognitive and behavioral changes with dementia develop in the later stages of the disease. The key structural neurodegenerative changes in the brain are loss of pigmented mesencephalic dopaminergic neurons projecting from the substantia nigra (pars compacta) to the neostriatum (consisting of caudate nucleus and putamen) and the accumulation of protein α-synuclein into inclusion bodies (*i.e.*, Lewy bodies) in neurons. Therefore, less dopamine is released into the neostriatum. Lewy bodies are the hallmark of the idiopathic form of PD. The distribution of Lewy bodies throughout the parkinsonian brain varies from one individual to another and is often reflected in the expression and degree of clinical signs of each individual patient with PD. The diagnosis of PD is based mainly on the presence of clinical signs (*i.e.*, parkinsonism), their response to specific treatment and confirmed with functional neuroimaging [10,16].

The essential change in brain function of patients with PD is the result of a reduced dopaminergic signaling in the basal ganglia with secondary changes in the release of neurotransmitters throughout the brain. Substitution treatment with levodopa (*i.e.*, L-dopa, l-dihydroxyphenylalanine), the natural dopamine precursor, is the gold standard for the initial treatment of PD [10,16]. The antiparkinsonian effect of levodopa in the brain is mediated primarily by the stimulation of postsynaptic D_2_ receptors. The conversion of levodopa to dopamine occurs in the presynaptic dopaminergic nerve endings. Therefore, an intact presynaptic apparatus is essential for levodopa to have an effect.

**Figure 1:**
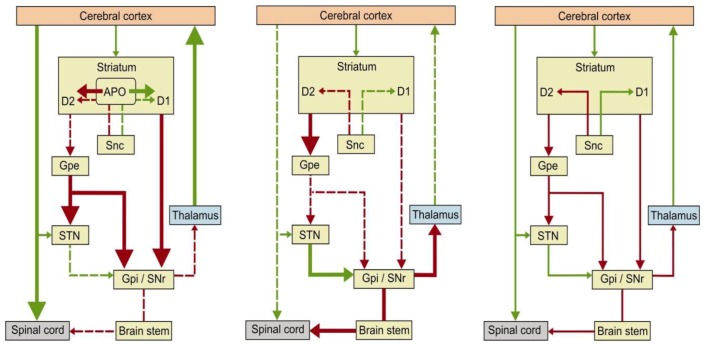
Activity of the major connections in the basal ganglia in patients with PD after APO medication (left); in untreated PD patients (center) and in healthy subjects (right) [10,18]. The green lines denote excitatory pathways; the red lines denote inhibitory pathways. A thick line denotes increased activity and a dotted line denotes a decreased activity. APO (apomorphine); D_2_, D_1_ (D_1_ and D_2_-like dopaminergic receptors); Gpe (external segment of globus pallidus); Gpi (internal segment of globus pallidus); Snc (compact segment of substantia nigra); SNr (reticular segment of substantia nigra); STN (subthalamic nucleus).

The antiparkinsonian effectiveness of APO is similar to that of levodopa [10]. APO readily diffuses across the blood-brain barrier, unlike levodopa that has to bind to a saturable, carrier-mediated transport system [17]. In contrast to levodopa, APO is not concentrated in the presynaptic dopaminergic endings and its beneficial motor effects in patients with PD are not dependent on the presence of functional presynaptic nerve endings since it does not require decarboxylation to be activated [2]. The suggested APO mechanism for improvement of motor symptoms in patients with PD [10] is the drug’s attenuation of thalamic inhibition by the internal globus pallidus with subsequent activation of the cerebral cortex (Figure 1). Of note is that in therapeutic concentrations APO reduced the firing rate in the subthalamic nucleus and the internal globus pallidus but not in the external globus pallidus in patients with PD [18]. APO’s dyskinetic effect is probably mediated by an excessive activation of the afferents to the centromedian-striatopallidal or pallidal-pedunculopontine pathways [19].

APO is used to assess PD patients’ responsiveness to levodopa or a dopamine agonist, as a rescue therapy for sudden off-periods or as a continuous infusion for treatment of motor fluctuations or dyskinesia. PD patients’ response to levodopa or dopamine agonists is evaluated by the APO challenge test. This test has an 80% predictive ability to foretell dopaminergic responsiveness and to clinically diagnose PD across all stages [10]. Long-term use of levodopa is associated with adverse motor events-motor fluctuations and dyskinesia. The term “motor fluctuations” is used when the duration control of PD motor symptoms with levodopa becomes shorter than expected. Motor fluctuations progress from early wearing-off to predictable off-periods to unpredictable off-periods and finally to yo-yoing (*i.e.*, the on-off phenomenon). The “early wearing-off” symptom is caused by the progressive reduction in the number of dopamine producing nerve cells that has reached the stage where the reduced capacity to produce dopamine cannot be compensated with levodopa. The timing of off-periods first occurs when the next dose of levodopa is due, but becomes less predictable (unrelated to levodopa brain concentration) with the progression of PD. Levodopa induced dyskinesia (involuntary movements of the limb, trunk or facial muscles, e.g*.*, chorea, tic or myoclonus) is attributed to the increased sensitivity of brain cells concerned with motor control (an adaptation to chronically reduced levels of dopamine) when dopamine concentration is transiently increased after taking a dose of levodopa [10,16].

Treatment with levodopa for more than five years will lead to the development of motor fluctuations in more than half of patients with PD [20]. After 15 years of disease almost 90% of patients with PD develop motor fluctuations [21]. These motor fluctuations can significantly reduce the patient’s quality of life [10,16]. Continuous infusion of levodopa or dopamine agonists can reduce the frequency of motor fluctuations presumably by avoiding oscillations in dopamine brain concentrations. APO, a dopaminergic agonist, delivered in intermittent *s.c.* injections or as a continuous *s.c.* infusion has been used in patients with advanced PD for the treatment of persistent and disabling motor fluctuations [22,23,24]. APO treatment is restricted only to patients with advanced PD due to the high cost of the drug, the necessary concomitant treatment for prevention of side-effects and the need for *s.c.* administration as the only practical long-term route of administration [25].

Long term use of APO, for treatment of patients with PD, leads to a progressive loss of response, *i.e.*, tolerance. Fortunately, the time interval between APO doses, necessary to prevent tolerance is brief, at least four hours [26,27]. APO administration can lead to side effects which tend to increase in frequency with the duration of drug use. Deleu and coworkers [10] summarized the adverse drug reaction profile and reasons for dropout in the clinical trials with intermittent *s.c* and continuous *s.c* infusions of APO. Some of the most frequent side effects of long-term APO therapy were orthostatic hypotension (19% and 5%), nausea (17% and 10%), fibrotic nodules at the site of APO infusion (11% and 70%) and sedation (6% and 23%) for intermittent *s.c.* and continuous *s.c.* infusions respectively [10]. Dopaminergic side effects of APO therapy such as orthostatic hypotension and vomiting can be attenuated by peripherally acting D_2_ receptor blocking agent domperidone, which does not penetrate the blood-brain barrier but has access to the chemoreceptor trigger zone [10]. Not all APO side effects are mediated by APO’s action on D_2_ receptors. For example, nausea is mediated by APO’s interaction with dopamine receptors and probably also with opiate receptors. It has been reported that APO’s analgesic effects can be attenuated by naloxone [28] which does not interact with dopamine receptors. Thus APO elicited nausea is attenuated by the actions of D_2,3_ receptor blocking agents (e.g*.*, domperidone) and μ-opioid receptor competitive antagonists (*e.g.*, naloxone) [29]. Compared to other dopamine agonists, the long term use of APO is less likely to induce the dopamine dysregulation syndrome characterized by hypersexuality or addiction to medication and gambling [30].

Several recent studies compared the long term treatments of advanced PD with *s.c.* APO administration, deep brain stimulation (DBS) of the subthalamic nucleus or duodenal levodopa infusion [16,22,23,24,31]. All three treatments improve the patient’s quality of life (*i.e.*, reduce the frequency and duration of levodopa induced dyskinesias) and all three require patient support from a movement disorder clinic with access to surgery. Treatment with duodenal levodopa infusion is preferable to DBS for patients who have contraindications for surgery (e.g., age over 70, severe depression). The cost of the drug for duodenal levodopa infusion is very high, comparable to APO [16]. However, cost benefit analysis shows that successful APO treatment reduces the need of visits and admissions to hospital and allows for the reduction in the dose of levodopa [32,33]. The effects of DBS are best when DBS stimulation is combined with medication adjusted to the patient’s needs [23,34]. However, not all of the signs of advanced PD improve to the same degree. For example, with DBS treatment, the least improved signs of motor fluctuations are speech disabilities (hypophonia and dysarthria) and freezing of gait [22]. A 5-year prospective assessment of patients with advanced PD that were treated with *s.c.* APO infusion or DBS reported that both therapies decreased daily off-time but only DBS reduced the duration and severity of dyskinesia. At present, there is no consensus on the best treatment for advanced stages of PD [22].

## 3. Apomorphine Treatment in Erectile Dysfunction

Erectile dysfunction (ED) is defined as the persistent inability to achieve and/or maintain an erection sufficient for satisfactory sexual intercourse [35]. ED affects up to 50% of men between ages 40 and 70 [36]. Treatment with specific phosphodiesterase 5 inhibitors is effective provided there is an adequate local release of the principal mediator of erection—NO. Currently, new potential treatments for low NO related ED with quanylyl cyclase activators or stimulators, rho-kinase inhibitors or sodium nitrite are being investigated [36].

ED has organic or psychogenic causes, and often is a combination of both. Less than 40% of patients are estimated to have psychogenic ED; the organic causes of ED are subdivided into vascular, neurologic, hormonal or smooth muscle abnormalities [37,38,39]. It has been suggested that some psychogenic cases of ED are misdiagnosed because the organic cause could not be identified [40]. The line between a psychogenic and organic cause of ED can be difficult to establish since patients with stress disorders and depression can have an increased adrenergic tone, which affects also their corpus cavernosum smooth muscle tissue responsiveness. Well known risk factors for ED are heavy smoking, alcohol abuse, hypercholesterolemia, diabetes, advanced age and numerous medications (e.g., antipsychotics, anticholinergics, antihypertensives, antidepressants…) [40,41].

**Figure 2:**
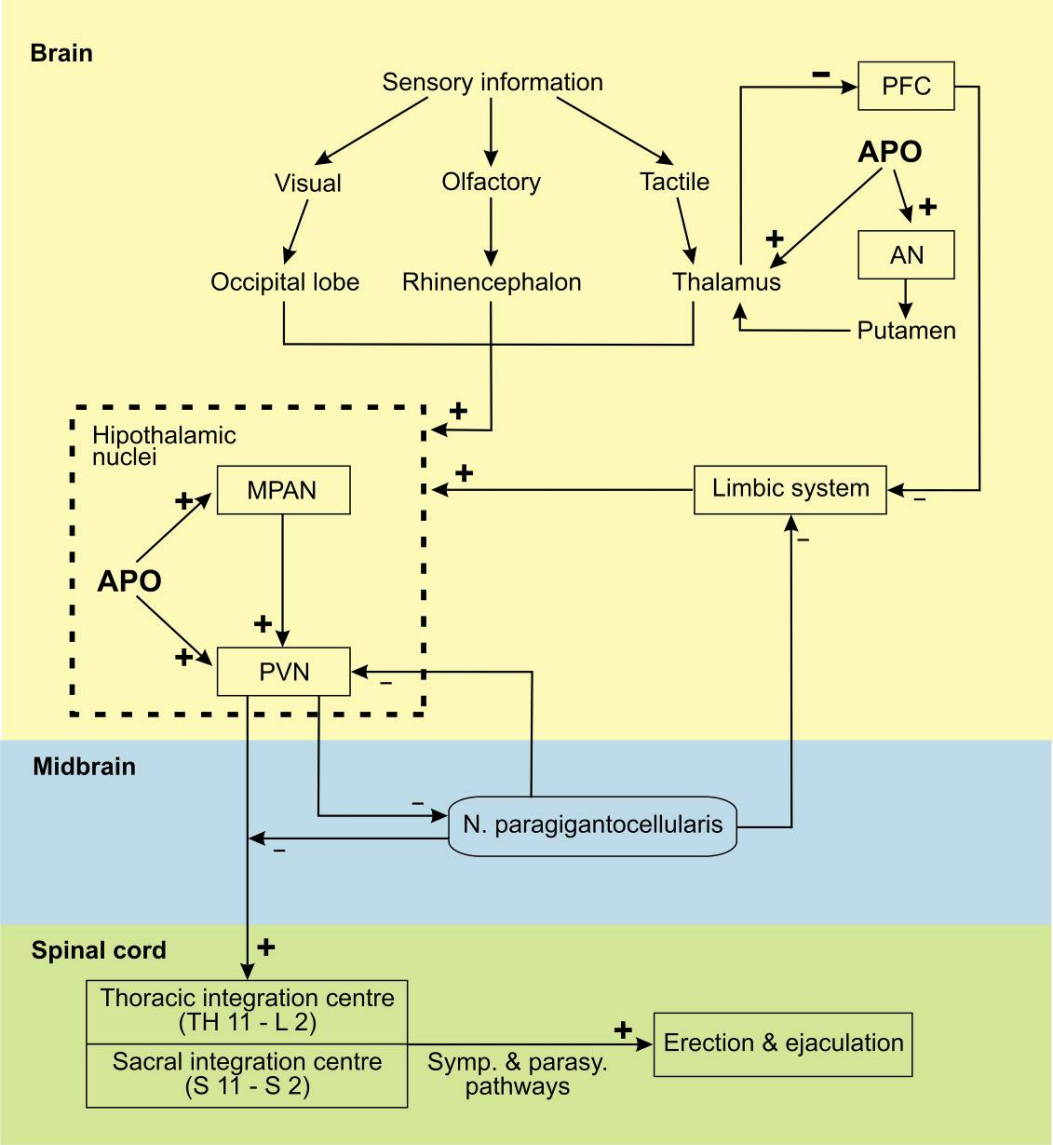
Central pathways controlling erection and ejaculation [36,42,43]. AN (accumbens nucleus); APO (apomorphine); MPAN (medial preoptic area nucleus); PFC (prefrontal cortex);PVN (paraventricular nucleus); **+** (accentuated activity); − (attenuated activity). Central dopaminergic neurons form synapses in the PVN and MPAN and dopaminergic neurons project from the hypothalamus to the spinal cord. APO promotes erection by acting mainly on the D_2_-like receptors in the central nervous system. The effect of APO on erection and ejaculation is shown with bold (**+**) or (**−**) symbols.

Peripheral and central mechanisms, reviewed in [36,42,43], are responsible for the regulation of erection. Briefly, erection occurs after processing of sexual stimuli (visual, olfactory, tactile and imaginative) in the brain (Figure 2). These stimuli are integrated and processed in the paraventricular nucleus (PVN) and in the medial preoptic area nucleus (MPAN) into penile erectile responses. The MPAN receives stimuli from the occipital lobe, rhinencephalon, thalamus and limbic system and integrates the received information into sexual motivation and copulatory motor programs. The PVN initiates the erection and ejaculation nerve signals. Dopaminergic neurons from the PVN run through the midbrain, where the proerectile signals can be attenuated by the nucleus paragigantocellularis (PGCN), if an erectile response is inappropriate, and terminate within the two main spinal sites of integration for erectile function (located at spinal segments T11-L2 and S2-S4). The thoracic and sacral spinal erection centers elicit reflex responses by integrating information from the periphery (from spinal sympathetic and parasympathetic afferents) and from the supraspinal level. Thus dopamine participates in the regulation of the autonomic and the somatic components of the penile reflex [43]. From the above mentioned spinal centers, the erectile signals are conveyed to the periphery by the parasympathetic and sympathetic autonomic neurons. Activated parasympathetic nerve endings release NO and acetylcholine. NO promotes muscle relaxation in the corpus cavernosum and erection while noradrenalin, released from the sympathetic nerve endings, promotes smooth muscle contraction and inhibits erection. Other substances, that also modulate the CNS control to stimulate erection, are oxytocin, adrenocorticotropic hormones, NO and L-glutamate [43]. Serotonin and GABA inhibit penile erection [43]. Prolactin inhibits erection but the mechanism can be either central or through a direct effect on corpus cavernosum smooth muscle contractility [43]. Peripherally, the functional state of the penis is determined by the balance of factors [43] that promote erection—Vasodilatation agonists (e.g*.*, NO, prostaglandins E and D or vasoactive intestinal polypeptide) or detumescence—vasoconstrictor agonists (e.g*.*, noradrenalin, endothelin 1, angiotensin II, thromboxane A_2_ or prostaglandin F_2α_).

A brief description of the mechanism of erection is as follows. Psychological and/or physical stimulation of the penis of a healthy male elicits the release of NO from the nerve endings in the corpus cavernosum. NO dilates the cavernous arteries thus increasing blood flow into the penis. The increased blood flow shearing forces stimulate the endothelial cells lining the lacunar spaces to produce and release more NO. The production of NO is regulated through the nitric oxide synthase enzyme and the enzyme’s activity is modified by many endogenous substances for example HDL, LDL or androgens [41]. The increased NO concentration relaxes the corpus cavernosum smooth muscle tissue, increasing its blood volume and pressure by trapping of the incoming blood and simultaneously compressing the venous structures beneath the rigid tunica albuginea thus substantially reducing blood outflow and further increasing the rigidity of erection. A further pressure increase is provided by contraction of the ischiocavernosus muscles [36,40,44].

The intracellular second messengers that mediate smooth muscle relaxation are cyclic adenosine monophosphate (cAMP) and cyclic guanosine monophosphate (cGMP). Intracellular NO increases the production of cGMP by stimulating the guanylyl cyclase enzyme. Intracellular concentration of cAMP is increased by increased binding of prostaglandin E_1_ to cell membrane receptors that are coupled to the G-protein-adenylyl cyclase enzyme complex. cAMP and cGMP activate specific protein kinases, and these kinases phosphorylate specific proteins thus opening K^+^ channels, closing Ca^2+^ channels and sequestering intracellular Ca^2+^ into the endoplasmic reticulum. A fall in intracellular cytoplasmic [Ca^2+^] leads to smooth muscle relaxation. Phosphodiesterase inhibitors (specific—e.g., sildenafil; or nonspecific—e.g., papaverine) reduce the breakdown of cGMP by phosphodiesterase 5 [36,40,43,44].

APO has long been known as an erectogenic agent but only the development of a sublingual (*s.l.*) formulation, that maintained the erectogenic function while decreasing adverse events, enabled its use in human medicine [45]. APO stimulates postsynaptic dopamine receptors (D_1_ and D_2_) in the hypothalamus and is effective as a precoital *s.l.* agent. A European multicentre study on 507 patients (254 received APO and 253 a placebo) with ED, evaluated the tolerability of *s.l.* APO in a forced dose-escalation regimen. The conclusion of the study was that the use of *s.l.* APO is safe and well tolerated by the patients. Adverse effects were not treatment limiting. The risk/benefit ratio analysis supported APO as a safe and effective alternative in managing ED [46]. The results of the European multicentre study were not supported by the results of another large study that examined the safety and use of APO, as a treatment of ED, prescribed in general practice in the UK [47]. The study cohort comprised 11.111 male patients with a median age of 61 years. Exposure information, during a 15 month interval, was gathered from dispensed prescription data for patients first prescribed APO. Maclennan and coworkers reported that the proportion of patients for whom APO was effective was low and a high proportion of patients stopped APO medication because they considered it to be “*without effect*”. The most often reported clinical adverse effects in this study were headache and nausea [47]. The effectiveness, safety and tolerability of sildenafil and APO were compared in a study involving 108 patients with ED. The conclusion of the study was that sildenafil was more effective in comparison to APO [48].

APO, a centrally acting modulator of sexual responses, could be considered as an alternative to sildenafil for patients with ED that do not respond to, or do not tolerate phophodiesterase type 5 inhibitors, provided there is no significant end-organ damage [43]. APO-induced brain modulation during sexual stimulation was studied with brain functional magnetic resonance (fMR) imaging. Compared to controls, patients with psychogenic ED had an increased activity in frontal limbic areas that was downregulated by APO [49]. Also, APO increased the activity of nucleus accumbens, hypothalamus and mesencephalon of patients with psychogenic ED [49]. In summary, due to APO modulation, the brain (fMR) image of patients with ED became similar to healthy controls. APO could also be useful for the treatment of female sexual arousal disorder. This possibility was explored by Caruso and coworkers [50] in a placebo controlled study. The efficacy and safety of daily *s.l.* intake of APO was investigated in premenopausal women affected by hypoactive sexual desire or sexual arousal disorder. The conclusion of the study was that daily *s.l.* intake of APO may improve the quality of sexual life of affected women [50].

## 4. Apomorphine Treatment in Alzheimer’s Disease

Alzheimer’s disease (AD) is the most common neurodegenerative disease in human characterized by a progressive and irreversible loss of cognitive functions [51,52,53]. The average life expectancy following the first diagnosis is approximately seven years, and less than 3% of individuals live more than 14 years after the first diagnosis [54,55,56]. Current experimental therapies are effective mainly as a preventive measure [57,58,59]. The cause of AD is not well understood, but the consensus is that the two major changes of brain morphology are intracellular neurofibrillary tangles (NFTs) and extracellular senile plaques (SPs) [60].

### 4.1. The Role of APP in AD

In human, the amyloid precursor protein (APP) is a single-pass transmembrane protein with a large transmembrane domain. The physiological significance of APP is not fully understood although APP production in the mature, normal CNS is substantial [53]. APP is produced in nerve cells and has a fast turnover rate [61]. After processing in the endoplasmic reticulum and Golgi apparatus, APP can follow two intracellular pathways. It can be transported by the fast axonal transport to synaptic terminals where it is inserted into the synaptic cell membrane [62] or inserted into a cell body endosomal compartment. 

Attached to the cell membrane, APP can be proteolyzed by two competing pathways that generate either senile plaque-promoting byproducts or non-senile plaque-promoting byproducts. The enzyme α-secretase breaks down mature APP into a soluble peptide APPα and an 83-amino acid membrane bound C-terminal fragment (83-CTF). The soluble APPα diffuses into the extracellular space and the 83-CTF is further proteolyzed into a p3 peptide. The alternative, senile plaque promoting pathway, involves first the breakdown of APP with β-secretase—generating a soluble APPβ peptide and a 99-amino acid membrane bound C-terminal fragment (99-CTF) − and then proteolysis of the 99-CTF with γ-secretase into a APP intracellular domain fragment (APP-ICD) and a neurotoxic 37–44 amino acid Aβ peptide.

An alternative fate for the membrane bound APP is its reinternalisation into endosomes containing the β- and γ-secretases leading to the intracellular production of Aβ. This fraction of the Aβ peptide is exported to the extracellular space (via vesicular transport) or degraded in lysosomes. [53]. Reinternalisation of membrane bound APP seems to be an important pathway for the production of Aβ since 80% of Aβ release into the extracellular space is blocked by inhibiting endocytosis [62]. Under native conditions, APP is proteolyzed by β-secretase located predominantly in endosomes [63]; γ-secretase activity is present on the cell surface and in the endosomes [64,65]. α-secretase proteolysis of APP occurs mainly on the cell surface, but there is also some α-secretase proteolysis of APP in the Golgi apparatus [53].

Accumulation of soluble and unfolded monomers of Aβ peptides promotes their conformational change, formation of oligomers and aggregation of fibrils. The final stage of Aβ peptide assembly in the extracellular space is the formation of amyloidal plaques in the gray matter of the brain. Aβ42 monomers with intermediate conformations and Aβ42 oligomers are assumed to be the most potent neurotoxins while the amyloid plaques are supposed to be relatively inert [66,67]. Aβ accumulation and processing in the extracellular space is accelerated by chronic inflammation, as a result of Aβ-linked cell death, and also by directly eliciting an inflammatory response of astrocytes and microglia by increasing the expression of inflammation associated proteins [68,69]. In addition, inflammation leads to a local increase in the concentration of reactive oxygen species (ROS), H_2_O_2_ and O_2_^−^, that oxidize prostaglandins forming F_2α_-isoprostanes. H_2_O_2_ and F_2α_-isoprostanes accelerate aggregation of Aβ [66,70].

The presence of extracellular Aβ42 leads to an increase in intracellular Aβ42 [60,71,72]. Intracellular accumulation of Aβ42 promotes H_2_O_2_ induced, p53 mediated apoptosis [73,74,75,76]. High resolution 3D images of brain samples of AD transgenic mice suggest that intraneuronal Aβ42 is concentrated at the distal neurites and the synapses. This local intracellular concentration of Aβ42 could contribute to the destruction of distal neurites and synapses leaving a surviving nerve cell with a reduced number of neurites and synapses and with adjacent extracellular accumulations of Aβ42 [77]. The local, increased extracellular Aβ42 concentration could propagate the Aβ42 related neurotoxic effect to neighboring nerve cells and also be further shaped into less neurotoxic amyloid plaques by the concomitant inflammatory process [60,78,79,80].

Patients with AD can accumulate senile plaques in their brains for decades before the onset of the first symptoms of cognitive impairment [81]. There are at least two possible, and non-excluding, explanations for the lack of correlation between Aβ plaques and cognitive impairment in patients with AD: either person-to-person differences in the ability of inflammatory cells to effectively remove Aβ plaques from the brain [60] or the high neurotoxic properties of Aβ42 oligomers that precede Aβ plaque formation.

### 4.2. The Role of Tau in AD

Tau is a phosphoprotein that interacts with tubulin to promote tubulin assembly into microtubules and also promotes microtubule stability. Tau is abundant in neurons of the CNS and also expressed at very low levels in astrocytes and oligodendrocytes.[82]. Tau activity is concentrated at the distal end of axons, thus promoting stability and flexibility of axon terminals. Kinases and caspases phosphorylate tau thus disrupting the microtubules; their actions on tau are opposed by phosphatases [53,83,84]. The degree of tau phosphorylation normally decreases with age due to the increased activity of phosphatases. Tau hyperphosphorylation is promoted by Aβ42 (via activation of kinases and caspases) and by serine protease inhibitor α_1_-antichymotrypsin ACT [68,85,86,87].

An important contributing factor to the development of AD could be oxidative stress—an imbalance between the production and inactivation of ROS—that is supposed to contribute to age-related changes in the brain [88]. Oxidative stress can increase Aβ levels (by a positive feed-forward loop between the β- and the γ-secretase that requires the JNK/c-jun signaling pathway) and tau phosphorylation in cell culture and in animal models, including AD animal models [89,90,91]. The link between oxidative stress and AD has been used to explain the increase in the frequency of AD with age [92].

### 4.3. APO As a Potential Treatment of AD

The potential, therapeutic effects of APO were studied in the triple transgenic AD mouse model 3xTg-AD and on cultured cells SH-SY5Y [93]. Himeno and coworkers reported that APO treatment of triple transgenic AD mouse improved short-term memory and led to significant decreases in intraneuronal Aβ and hyperphosphorylated tau. Untreated 3xTg-AD mice develop intraneuronal Aβ accumulation and memory disturbances before extracellular Aβ deposition. Aβ42 was detected in brain tissues of untreated mice but not in brain tissues of APO treated mice. APO treatment did not alter the level of Aβ40 measured in extracts of brain tissues. In cell cultures, APO promoted degradation of intracellular Aβ40 and Aβ42 presumably by increasing the activity of proteasomes and of insulin-degrading enzyme. Also, APO protected against H_2_O_2_ toxicity a finding that has been confirmed in experiments with a human neuroblastoma cell line [94]. The activities of γ-secretase and of the zinc metalloendopeptidase neprilysn were not significantly affected by APO treatment [93]. A dose dependent effect was observed with APO treatment since 20 mg/kg and 15mg/kg APO injections in mice were less effective on memory function than 5 mg/kg APO injections, although the intraneuronal Aβ and phophorylated tau content were decreased at higher doses.

**Figure 3:**
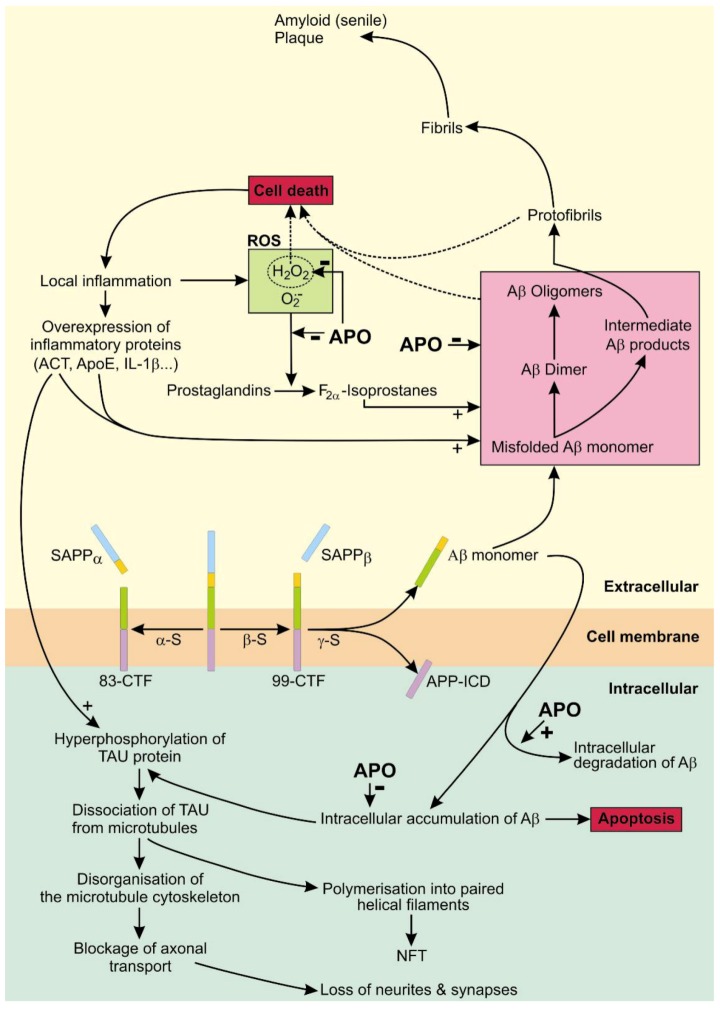
The non-amyloidogenic and amyloidogenic metabolic pathways of amyloid precursor protein (APP) [6,59,66,89,90,91,93,94,97,98,99,100,101]. APO (apomorphine); SAPPα (soluble peptide APPα); SAPP_β_ (soluble peptide APP_β_); Aβ (Aβ42 peptide); α-S (α-secretase); β-S (β-secretase); γ-S (γ-secretase); CTF-83 and CTF-99 (83 and 99-amino acid membrane bound C-terminal fragment), NFT (neurofibrillary tangles); APP-ICD (APP intracellular domain); ROS (reactive oxygen species); **+** (accentuated activity); − (attenuated activity). The effect of APO is shown with bold (**+**) or (**−**) symbols.

APO is a well-known non-selective dopamine agonist and has also a potent antioxidant effect. Therefore, the reported beneficial effects of APO on AD could be mediated by dopamine receptor-dependent or dopamine receptor-independent mechanisms. Activation of dopamine D_4_ receptors was reported to inhibit oxidative stress-induced nerve cell death [95]. The synaptic activity reduces intraneuronal Aβ and protects against Aβ-related synaptic changes by a mechanism involving the protease neprilysin [96]. However, a dopamine receptor-dependent mechanism seems not to be involved since memory function could not be improved by pramipexole, an alternative dopamine agonist acting on D_2_, D_3_ and D_4_ receptors [93] and APO treatment did not increase neprilsyn activity [93]. Therefore, it seems more likely that the protective, anti AD effects of APO are mediated by dopamine independent pathways (Figure 3).

The hypothetical, AD-protective dopamine-independent effects of APO, supported by direct or indirect experimental evidence, are increased intracellular degradation of Aβ40 and Aβ42 [93], protection against oxidative stress [6,89,90,91,93,97,98] and a reduced oligomerisation and subsequent aggregation of Aβ42 into fibrils that form senile plaques [66,67,70,99,100,101]. Increased intracellular degradation of Aβ could prevent the tau protein hyperphosphorylation and preserve the nerve cells neurites and synapses. Preservation of interneuronal connections could enable a sufficient synaptic activity to reduce intraneuronal Aβ and to protect against Aβ-related synaptic alterations and decrease of synaptic transmission. A reduced breakdown of the distal axonal projections would prevent the release of Aβ42 into extracellular space thus (a) reducing the spread of its toxic effect to neighboring cells; (b) reducing the Aβ42-elicited inflammatory response of astrocytes and microglia in the CNS and (c) reducing the amount of extracellular Aβ42 aggregation into fibrils and of formation of oligomers, both that are highly neurotoxic. By reducing oxidative stress, APO could also prevent aggregation of Aβ fibrils precipitated by ROS. The overall effect of the aforementioned APO-induced changes could preserve nerves, nerve connections and ultimately cognitive function.

The potential of APO for treatment of AD will be further enhanced by supportive data from independent laboratories. Assuming the exciting results reported by Himeno and coworkers will be confirmed, there are many questions that have to be answered before APO can be certified for treatment of AD. The APO doses that had an anti-AD effect in cell cultures and in mouse models [93] were about 50–100 times higher than the APO doses for treatment of PD in human patients. For example, the mean dose for the APO test in PD is 3 mg per adult [102]. However, the minimal effective dose for an APO effect in the triple transgenic AD mouse model was not determined and could be considerably lower than the dose used in this experiment. Therefore, an effective and safe dose for APO treatment of AD in patients has yet to be determined. It would be interesting to investigate if PD patients on continuous APO therapy have a lower incidence of AD-associated accumulation of fibrillar Aβ peptide in the brain in comparison to the PD patients on alternative therapies. If so, this would prove that a safe and effective dose for the treatment of AD in human is attainable. The practicalities of the long term use of APO are well known from the treatment of PD patients. At present, the only practical, long term mode of APO application (due to APO metabolism and the drug’s side effects) is by continuous *s.c.* infusion. This would suggest that APO treatment would be practical only for people with early sings of AD. Also, as mentioned previously, the cost of APO treatment by *s.c.* infusion is not insignificant but could be reduced by economies of scale if there is an increased demand.

## 5. Conclusions

The beneficial actions of APO treatment are dopamine-receptor mediated or non-dopamine-receptor mediated. The dopamine-receptor mediated effects are put to an effective use for the treatment of persistent and disabling motor fluctuations in patients with advanced Parkinson’s disease. The recently reported, non-dopamine-receptor mediated effects of APO treatment, in an animal model, suggest a new and potentially important therapeutic role for APO in the treatment of Alzheimer’s disease.
